# Risk Factors for Non-Occupational Carbon Monoxide Poisoning: Anshan Prefecture, Liaoning Province, China, 2011–2012

**DOI:** 10.1371/journal.pone.0129121

**Published:** 2015-06-12

**Authors:** Qiang Lu, Weiwei Lv, Jiang Tian, Lijie Zhang, Baoping Zhu

**Affiliations:** 1 Department of Neurology, Peking Union Medical College Hospital, Beijing, China; 2 Department of infectious disease control, Anshan prefecture Center for Disease Control and Prevention, Liaoning, China; 3 Department of infectious disease control, Liaoning Center for Disease Control and Prevention, Shenyang, China; 4 Field Epidemiology Training Program, Chinese Center for Disease Control and Prevention, Beijing, China; University of Missouri, UNITED STATES

## Abstract

**Background:**

Carbon monoxide (CO) poisoning can be fatal but is preventable. From October 2010 to February 2011, Anshan Prefecture reported 57 cases of non-occupational CO poisoning in District A, with two deaths. We conducted an investigation to identify risk factors and recommend preventive measures.

**Methods:**

We defined a possible case of non-occupational CO poisoning as onset of at least two of the following symptoms: fatigue, headache, dizziness, nausea, vomiting, cyanosis, loss of consciousness, coma, and shock from October 1, 2010, to February 28, 2011, in a resident of Anshan Prefecture with non-occupational exposure to CO poisoning. We defined a probable case as onset of at least one of the following symptoms: cyanosis, loss of consciousness, coma and shock, plus at least one of the following symptoms: fatigue, headache, dizziness, nausea, vomiting, among possible cases. A confirmed CO poisoning case was a possible case or probable case plus hemoglobin (Hb) CO higher than 10%. We searched for cases by reviewing medical records and records of hyperbaric oxygen tank usage. In a case-control investigation, we compared home heating practices of 30 case-persons and 120 control-persons who were individually matched to each case by neighborhood.

**Results:**

Overall, 56% (39/70) of case-patients’ households burned coal for home-heating. In the case-control investigation, 40% (12/30) of case-persons’ households compared with 5.8% (7/120) of control-persons’ households placed stoves in bedrooms (Mantel-Haenszel odds ratio [OR_M-H_] = 11, 95% confidence interval [CI] = 3.0–41); 53% (16/30) of case-patients’ households and 33% (40/120) of control-patients’ households did not extinguish the fire before sleeping (OR_M-H_ = 3.6, 95% CI = 1.1–12); 13% (4/30) of case-patients’ households and 3% (4/120) of control-patients’ households had not installed the ventilation pipe vertically (OR_M-H_ = 7.3, 95% CI = 1.0–56). Overall, 77% (23/30) of case-patients’ households and 39% (47/120) of control-patients’ households had at least one of those three risk factors (OR_M-H_ = 10, 95% CI = 2.5–40; population attributable risk percentage: 78%).

**Conclusions:**

Dangerous practices with coal-burning stoves inside the home accounted for the majority of CO poisoning incidents. Community health centers should provide instruction to and supervision of residents on proper installation and use of home heating stoves as well as inspection of installation.

## Introduction

Carbon monoxide (CO) is a colorless, odorless, tasteless, and nonirritating toxic gas produced by incomplete combustion of organic compounds [1]. Symptoms of CO poisoning are nonspecific. Mild exposures result in headache, myalgia, dizziness, or neuropsychological impairment [2]. Severe exposures result in confusion, loss of consciousness, or death [3]. Improperly installed heaters, motor vehicles, appliances that use carbon fuels, and most importantly household fires are the main sources for CO gas [1].

In China, CO poisoning cases are reported through the emergency public health event reporting system. In Anshan Prefecture, located in northeastern China where winters are bitterly cold, some residents that live in bungalows burn coal in stoves for home heating. From October 2010 to February 2011, the emergency public health event reporting system in Anshan Prefecture reported 57 non-occupational cases of carbon monoxide poisoning in District A, including two death (attack rate: 19/100000). In 91% of cases CO poisoning occurred as a result of household CO sources. The detailed causes included leakage of CO from coal-burning stoves, forgetting to turn off the natural gas valve, leaking gas cylinder used for cooking, and leaking gas-powered water-heater in the home. We conducted this investigation to identify risk factors for household sources of CO poisoning and recommend preventive measures.

## Materials and Methods

### Ethics statement

The study was approved by the Ethic Review Committee of Liaoning Center for Disease Control and Prevention. All study objects had signed an informed consent document prior to participation. All data analyzed were anonymized.

### Case definition

We defined a possible case of non-occupational CO poisoning as onset of at least two of the following symptoms: fatigue, headache, dizziness, nausea, vomiting, cyanosis, loss of consciousness, coma, and shock from October 1, 2010, to February 28, 2011, in a resident of Anshan Prefecture with non-occupational exposure to CO poisoning. We defined a probable case as onset of at least one of the following symptoms: cyanosis, loss of consciousness, coma, and shock, plus at least one of the following symptoms: fatigue, headache, dizziness, nausea, vomiting, among possible cases. A confirmed CO poisoning case was a possible case or probable case plus hemoglobin (Hb) CO higher than 10% (normal concentration: 2% for non-smokers and 5–9% for smokers [4]).

### Case finding

We searched for cases in all three hospitals designated to treat CO poisoning in Anshan Prefecture by reviewing the outpatient records, inpatient records, usage records of the hyperbaric equipment, and laboratory records. We also reviewed records in the emergency public health event reporting system to identify reported CO poisoning cases or events. We interviewed the case-patients about all sources of CO they were exposed to both inside and outside the home.

### Case-control study

We conducted a case-control study to identify risk factors for CO poisoning among residents living in bungalows who used coal-burning stoves for heating in the winter. Because this investigation focused on household-level risk factors, if a household had multiple case-patients, we selected only one. For each case-patient, we randomly selected four individually-matched neighborhood controls from households without any suspected cases; one control was selected in each household. We interviewed the case-persons and control-persons or their family members to collect information regarding home heating practices, e.g., type of stove used, type of fuel used (e.g., block coal, powdered coal, charcoal), design of the chimney, location of the stove in the household, how frequently the ventilation pipes was cleaned, how the ventilation pipe was installed, and whether the stove fire was extinguished before sleeping.

### Statistical analysis

We used univariate conditional logistic regression to estimate the matched odds ratios (OR_match_) and 95% confidence intervals (CI) associated with various risk factors, and multivariable conditional logistic regression to control for confounding and to calculate the adjusted odds ratios (OR_adj_). We used the OR_adj_ and the exposure percent in the control group associated with each risk factor to calculate the population attributable risk percent (PARP) for each risk factor[5].

## Results

In total, we identified 72 cases (54 possible, 12 probable and 6 confirmed) in 67 households; 62 of the 67 households had one case and five had two cases. The median age of the case-patients was 47 years (range: 12–83 years); 56% (40/72) of the case-patients were males. Cases occurred in three out of four districts in Anshan Prefecture; 97% (70/72) of the cases occurred in District A where the bungalows were concentrated; District B and C each had one case. Therefore, we focused our investigation on cases in District A.

Of the 70 case-patients in District A, the clinical symptoms included fatigue (43%), dizziness (39%), headache (37%), nausea or vomiting (33%), confusion (14%), and coma (11%). Two of the case-patients died (case-fatality rate: 2.9%). Interviews of the case-persons revealed that the majority of the CO poisoning incidents (56%, 39/70) were caused by leakage of CO from coal-burning stoves used for home heating (Table 1).

**Table 1:** Causes of non-occupational carbon monoxide poisoning: Anshan Prefecture, Liaoning Province, China, October 1, 2010, to February 28, 2011.

Cause of CO poisoning	Cases (n = 70)	Percent (%)
Leakage of CO from a coal-burning stove inside the home	39	56
Forgetting to turn off the natural gas valve	13	19
Leakage of CO from coal-burning stove in sealed sun room	7	10
Leaking gas cylinder used for cooking	7	10
CO generated from hot-pot stoves	2	2.9
Leaking gas-powered water-heater	2	2.9

The 39 case-patients whose cause of CO poisoning was the use of coal-burning stoves inside the home lived in 36 bungalows. We enrolled 30 case-patients (from 30 different households) in our investigation; the other six households had either moved away at the time of our investigation or could not be reached due to lack of detailed contact information.

The 30 households all used coal-burning stoves for home-heating. None of the 30 case-patients’ households had installed CO detectors. None of the 120 control households had installed CO detectors. The case-control investigation showed that 40% (12/30) of case-patients’ households installed a stove in the bedrooms compared to 5.8% (7/120) of control-persons’ households (OR_match_ = 11, 95% CI = 3.5–37); 97% (29/30) of case-patients’ household and 76% (91/120) of control-persons’ households burned block coal (OR_match_ = 9.3, 95% CI = 1.2–72); 13% (4/30) of case-patients’ households and 3.3% (4/120) of control-persons’ households installed non-vertical ventilation pipe (OR_match_ = 7.0, 95% CI = 1.1–34); 80% (24/30) of case-patients’ household and 67% (80/120) of the control-persons’ households never cleaned the ventilation pipes (OR_match_ = 4.2, 95% CI = 1.0–22); 53% (16/30) of case-patients’ households and 33% (40/120) of control-persons’ households did not extinguish the fire before sleeping (OR_match_ = 2.7, 95% CI = 1.1 = 6.9). The multivariable analysis showed that placing the stove in the bedroom (OR_adj_ = 11, 95% CI = 2.0–41), installing non-vertical ventilation pipes (OR_adj_ = 7.3, 95% CI = 1.0–56), and not extinguishing the fire before sleeping (OR_adj_ = 3.6, 95% CI = 1.1–12) were significant risk factors for CO poisoning (Table 2). Other factors, including ventilation pipes without joints, ventilation pipes made of brick, sealing off the chinks around the windows to prevent the wind from blowing in, lack of a ventilation fan, and using brick stoves, were not significantly associated with CO poisoning.

**Table 2:** Risk factors for non-occupational CO poisoning: Anshan Prefecture, Liaoning Province, China, October 1, 2010, to February 28, 2011.

Risk factor	# cases (N = 30)	# controls (N = 120)	% Cases	% Controls	Matched OR (95% CI)	OR_adj_ (95%CI)
Placement the stove in the bedrooms	12	7	40	5.8	11(3.5–37)	**11(3.0–41)**
Installing non-vertical ventilation pipes	4	4	13	3.3	7.0(1.1–34)	**7.3(1.0–56)**
Did not extinguish the fire before sleep	16	40	53	33	2.7(1.1–6.9)	**3.6(1.1–12)**
Burned block coal	29	91	97	76	9.3(1.2–72)	5.1(0.65–40)
Did not clean ventilation pipes	24	80	80	67	4.2(1.0–22)	3.8(0.32–45)
Stove type						
Mud stove	4	6	13.3	5.0	3.0(0.77–12)	
Iron stove	13	44	43	37	1.3(0.59–2.9)	
Brick stove	13	70	43	58	0.54(0.23–1.3)	
Ventilation pipes had no joints	22	78	73	65	1.8(0.61–5.5)	
Ventilation pipes made of brick	10	34	33	28	1.4(0.49–4.2)	
Ventilation pipes made of iron	11	34	37	28	1.7(0.62–4.5)	
Ventilation pipes made of concrete	5	23	17	19	0.78(0.22–2.8)	
Ventilation pipes of pottery	10	34	33	28	1.4(0.49–4.2)	
Sealed off chinks around windows to prevent wind from blowing in	19	78	63	65	0.92(0.38–2.2)	
Did not use a ventilation fan	14	61	47	51	0.82(0.34–2.0)	
Sealed joints of chimney tunnel	8	41	27	34	0.58(0.19–1.7)	
Checked chimney tunnel routinely	4	24	13	20	0.45(0.11–1.93)	
Added coal into stove before sleeping	11	53	37	44	0.67(0.26–1.7)	

The population attributable risk percentages (PARP) were 37% for installing a stove in the bedrooms, 17% for having a non-vertical ventilation pipe, and 46% for not extinguishing the fire before sleeping. When we examined those three risk factors combined, 77% of the case-patients’ households compared with 39% of the control-persons’ households had at least one of the three risk factors (OR_M-H_ = 10, 95% CI = 2.5–40), yielding a PARP of 78%.

## Discussion

About half of the world’s households depend on biomass and coal for cooking and heating; most of these are found in developing countries, where poor households generally use open fires or inadequately vented stoves [6–7]. In Anshan Prefecture, China, residents who live in the bungalows mainly use stoves for home heating in winter. Of the 70 case-patients in District A, the majority of the CO poisoning incidents (56%) were caused by leakage of CO from coal-burning stoves used for home heating. That means CO poisoning could be greatly decreased if the stoves were installed and used correctly. So, we conducted a case-control study to identify the risk factors for CO poisoning among the residents who used coal-burning stoves for heating. We identified three preventable risk factors involved in the installation and use of coal-burning stoves for home heating. Previous literature in China had shown that use of a stove could increase the risk of CO poisoning [8], but in our investigation, in addition to the placement of stoves in the bedroom that had previously been shown to be a risk factor, we also found having a non-vertical chimneys and not extinguishing the fire before sleeping were risk factors for CO poisoning. In this prefecture, this problem would continue to exist if not identified and resolved. The results of this study could be applied not only in this prefecture but also anywhere in the world where stoves were used to greatly reduce CO poisoning caused by coal-burning stove. In our case, CO poisoning is a significant, yet completely preventable, cause of death and severe illness in northern China. However, applied research on locality-specific risk factors had rarely been conducted. Therefore, our study raised the awareness of this important public health, identified risk factors in this area and informed public health actions.

Previous investigations also showed that keeping windows closed is a risk factor for CO poisoning [8, 9]. However, we were unable to investigate this factor because, with average winter temperate of -10°C in Anshan Prefecture, doors and windows in the bungalows are universally closed at night in the winter. In this region, people usually seal windows which means sealing off the chinks around the windows when the windows were closed to prevent wind from blowing in through the chinks to maintain warmth in the room. Yet sealing the windows was not shown as a risk factor in this study. Use of mud stoves for home heating had been found to be a risk factor for CO poisoning in Hunan Province in southern China [8]. The mud stoves had no chimneys. When they are used in the bedrooms, CO gas can easily leak out of the top and bottom of the stove, causing CO poisoning [8]. In Anshan Prefecture, although mud stoves were used, they usually had chimneys. But if the chimneys were installed non-vertically, the risk of CO poisoning was increased. The gas in chimneys that are installed horizontally usually discharges slowly compared to the gas in chimneys that are installed vertically out of the room.

Many residents in Anshan Prefecture used stoves for home heating, especially those living in bungalows. Our studies showed that factors related to improper installation and use of those stoves accounted for over 78% of all CO poisoning cases, and these factors are all preventable.

The main limitation of this study was that several case-patients had moved away or their contact information was not available, resulting in the small study size.

The CDC definition for CO poisoning is: A case was defined as two or more symptoms consistent with CO poisoning (i.e., headache, nausea, diarrhea, dizziness, dry mouth, drowsiness, or vomiting) or CO poisoning diagnosed by a physician and a carboxyhemoglobin (COHb) level greater than 10% (normal concentration: less than 2% for nonsmokers, 5–9% for smokers) [10]. In our study, we formulated the case definition based on the symptoms of reported CO poisoning patients. Our case definition is similar to the CDC definition.

### Conclusion

In conclusion, we identified three preventable risk factors involving the installation and use of coal-burning stoves for home heating, which accounted for over three-quarters of CO poisoning cases.

### Recommendations

Based on the findings of this study, we suggest that the local government should conduct health education for local residents about the risk factors of CO poisoning, and provide instructions to the residents on the correct installation and use of home heating stoves. The local government should also consider providing free CO detectors to people living in bungalows and teaching them how to correctly install and use the detectors. The local government should also conduct periodic inspections of households living in bungalows before winter comes, and a few more times during the winter.
